# Preliminary exploratory research on the application value of oral and intestinal meta-genomics in predicting subjects' occupations–A case study of the distinction between students and migrant workers

**DOI:** 10.3389/fmicb.2023.1330603

**Published:** 2024-02-06

**Authors:** Shujie Dou, Guanju Ma, Yu Liang, Guangping Fu, Jie Shen, Lihong Fu, Qian Wang, Tao Li, Bin Cong, Shujin Li

**Affiliations:** ^1^College of Forensic Medicine, Hebei Medical University, Hebei Key Laboratory of Forensic Medicine, Research Unit of Digestive Tract Microecosystem Pharmacology and Toxicology, Chinese Academy of Medical Sciences, Shijiazhuang, China; ^2^Institute of Intelligent Medical Research (IIMR), BGI Genomics, Shenzhen, China; ^3^Hainan Tropical Forensic Medicine Academician Workstation, Haikou, China

**Keywords:** forensic microbiology, occupation estimation, metagenomic sequencing, random forest, recursive feature elimination

## Abstract

**Background:**

In the field of forensic science, accurately determining occupation of an individual can greatly assist in resolving cases such as criminal investigations or disaster victim identifications. However, estimating occupation can be challenging due to the intricate relationship between occupation and various factors, including gender, age, living environment, health status, medication use, and lifestyle habits such as alcohol consumption and smoking. All of these factors can impact the composition of oral or gut microbial community of an individual.

**Methods and results:**

In this study, we collected saliva and feces samples from individuals representing different occupational sectors, specifically students and manual laborers. We then performed metagenomic sequencing on the DNA extracted from these samples to obtain data that could be analyzed for taxonomic and functional annotations in five different databases. The correlation between occupation with microbial information was assisted from the perspective of α and β diversity, showing that individuals belonging to the two occupations hold significantly different oral and gut microbial communities, and that this correlation is basically not affected by gender, drinking, and smoking in our datasets. Finally, random forest (RF) models were built with recursive feature elimination (RFE) processes. Models with 100% accuracy in both training and testing sets were constructed based on three species in saliva samples or on a single pathway annotated by the KEGG database in fecal samples, namely, “ko04145” or Phagosome.

**Conclusion:**

Although this study may have limited representativeness due to its small sample size, it provides preliminary evidence of the potential of using microbiome information for occupational inference.

## 1 Introduction

In the field of forensic science, it is often essential to assess and forecast the personal identification details of specific individuals involved in a case. These details may include age, gender, height, facial and physical characteristics, medical condition, place of residence, and ethnicity. The identification of unidentified persons is an important aspect in daily forensic medicine works, including criminal investigation and disaster victim investigation. Unidentified human remains may cause many legal problems and affect the emotional level of the families of the victims. Therefore, forensic anthropologists play an important role in identifying the age, gender, descent, and height of human remains by creating biological archives (Gulhan et al., 2015). Currently, by analyzing the genetic information and physical traits, we can establish a connection between a particular phenotype and its corresponding genetic markers. This has led to significant interest in using molecular markers, specifically forensic DNA phenotypic inference, to identify human body characteristics. Examples include estimating age through DNA methylation (Xu et al., 2019; Naue, 2023) or telomeric DNA (Elmadawy et al., 2021), characterizing facial features using single nucleotide polymorphism (SNP) (Pospiech et al., 2022), and inferring age based on RNA (Rodriques et al., 2021; Wang et al., 2022).

However, in numerous real-life forensic cases, it proves challenging to acquire reliable human DNA data, and it becomes impossible to identify biological characteristics solely based on human DNA information. For example, saliva and fecal samples are commonly used as forensic materials, and extracting the identity information of the victim or suspect from these samples can be crucial for solving the case. However, it can be challenging to obtain the host DNA information from saliva and fecal samples. The human gut and mouth contain the largest microbial communities in the body, and by analyzing the abundant microbial DNA information, we can extract host information. In recent years, the introduction of massively parallel sequencing (MPS) technology has greatly improved the amount of sequencing data that can be used for forensic analysis (Oliveira and Amorim, 2018). Research has shown that both the human gut and oral microbiota are stable over the long-term and unique to individuals. This suggests that they could potentially serve as new markers for identifying human identities. In recent years, there have been several investigations that have demonstrated a connection between the microbial communities in the gut and oral cavity and various human traits. These traits include gender, body mass index, age, geographic region, race, and different diseases (Falony et al., 2016; Wu et al., 2018; Yang et al., 2019; DAngiolella et al., 2020; Aranaz et al., 2021; Chen et al., 2021; Salzmann et al., 2021; Gacesa et al., 2022). These human characteristics collectively impact the development of microbial communities in humans and contribute to their unique characteristics. Additionally, studies have shown that specific living habit can have influence on the characteristics. For instance, Liao et al. (2022) observed that the oral microbial α diversity of drinkers was significantly higher than that of non-drinkers; and Jia et al. (2021) found that the α diversity of oral microbiota in smokers was significantly higher than that of non-smokers.

The occupation of an identified person is a complex type of personal characteristic that is connected to various factors influencing the composition of microbes mentioned earlier. Making an accurate prediction of their occupation is also important for resolving certain cases. However, limited research has been conducted on how occupation affects the microbiota, despite the fact that various aspects of modern living can impact it. For example, a study by Hu et al. (2022) found differences in the composition of gut microbes between college athletes and healthy control individuals. The two groups showed variations in the proportion of microorganisms. At the genus level, the college athletes had higher levels of *Faecalibacterium* and *Bifidobacterium* but lower levels of *Bacteroides*. Additionally, studies have shown that different economic incomes can also influence the composition of intestinal microbiota (He et al., 2018; Widyarman et al., 2021; Gacesa et al., 2022; Zuniga-Chaves et al., 2023). Meanwhile, traditional amplicon-based sequencing methods have limitations in providing comprehensive microbial information, which has led researchers to focus more on species differences rather than the impact of microbial functional genes on hosts (Fricker et al., 2019). Metagenome sequencing, on the other hand, offers a more advanced approach as it does not rely on the isolation and culture of microorganisms, allowing for the study of difficult-to-culture and low-abundance microorganisms. It not only enables the analysis of species diversity in a sample but also provides insights into the functional diversity from a gene function perspective. In summary, there is a need for deeper studies on the intestinal and oral microbiota of individuals with different occupations, and metagenomic sequencing method can be helpful in this area.

Therefore, this study collected fecal and saliva samples from 50 individuals of two different occupations, laborers and students, for metagenomic research and analysis and studied the diversity and functional activity of microbial flora and other macro characteristics of different people from the perspectives of occupation, gender, smoking, and drinking. Moreover, machine learning method was used to deeply mine microbial metadata and build identity information prediction model, to explore the practical application value of microbiology in forensic medicine.

## 2 Materials and methods

### 2.1 Sample collection

This study included 50 healthy participants who had resided in the Hebei Province of China for an extended period (≥ 5 years). All participants were divided into three groups according to their gender and occupation, including 30 students from the same university (10 women and 20 men) and 20 male migrant laborers from the same community. The basic information, including age, BMI, education, and smoking and drinking status, of the study participants is shown in Table 1. All of the participants confirmed that they did not use any antibiotics for a period of 2 months prior to the collection of samples, and that they did not have any tumors or autoimmune diseases. Each participant signed an informed consent form before sampling, and the study was approved by the Medical Ethics Committee of Hebei Medical University, with the approval number 2023007.

**Table 1:** Basic information of the participants.

	**Female students (*N* = 10)**	**Male students (*N* = 20)**	**Male Laborers (*N* = 20)**
Age, mean(s.d.)	27.4 (2.32)	26.4(2.39)	52.6(12)
**BMI, n(%)**			
< 18.5	0 (0)	1 (5)	1 (5)
< 24	8 (80)	7 (35)	7 (35)
≥24	2 (20)	12 (60)	12 (60)
**Education, n(%)**			
Below high school	0 (0)	0 (0)	16 (80)
High school or above	10 (100)	20 (100)	4 (20)
**Smoking status, n(%)**			
Non-smoking	10 (100)	16 (80)	7 (35)
Smoking	0 (0)	4 (20)	13 (65)
**Drinking status, n(%)**			
Non-drinking	8 (80)	4 (20)	5 (25)
Drinking	2 (20)	16 (80)	15 (75)

From each participant, two types of samples were collected: i) 1ml of naturally flowing saliva, which was collected after a period of more than 2 h of fasting and water deprivation and ii) 2g of interrupted internal feces. In summary, 100 samples were collected, which were placed in separated sterile tubes and stored at -80°C until DNA extraction. These samples can be divided into six groups, which were labeled from three dimensions: (i) the gender of the origin individual (M: male or F: female); (ii) occupation of the origin individual (St: student or La: laborer); and (iii) the sample type (F: feces or S: saliva). For instance, saliva samples from female students were assigned into group “FStS”. For each sample, the number of them in the corresponding group would be labeled in their sample names, such as “FSt01S”.

### 2.2 DNA extracting and metagenomic sequencing

Total genomic DNA was extracted from the 100 samples using PowerSoil^®^ DNA Isolation Kit (Mo Bio Laboratories, Carlsbad, USA), according to the manufacturer's instruction. The quality and quantity of the extracted DNA were examined using Qubit dsDNA HS Assay Kit on a Qubit 3.0 Fluorometer (Life Technologies, Carlsbad, USA) and a 1% agarose gel electrophoresis.

Paired-end libraries with insert size of ~350 bp were prepared using a VAHTS Universal Plus DNA Library Prep Kit for Illumina (Vazyme Biotech, Nanjing, China). The library was sequenced on an Illumina NovaSeq 6000 platform using a 150-bp paired-end sequencing strategy (Biomarker Technologies Co., Ltd., Beijing, China). The Illumina raw sequence read data underwent base calling to generate two paired FASTQ files. Trimmomatic (Bolger et al., 2014) (version 0.33) was employed to evaluate the quality of the raw sequence reads. This involved trimming sequencing adapters, removing reads with a quality score below 20 over a sliding window of 50 base pairs and discarding reads with a sequence length of less than 100 base pairs. After the removal of adapters and low-quality reads, ***clean data*** is obtained for analysis.

The clean data were aligned to the human genome (*H. sapiens* GRCh38_release95) using bowtie2 (Langmead and Salzberg, 2012) (version 2.2.4), and any sequences that match with the human genome, as well as their complementary sequences, were removed. Then, the remaining metagenomic data were assembled using MEGAHIT (Li et al., 2015) (version 1.1.2), which makes the use of succinct de Bruijn graphs. During the assembling process, contigs with length of less than 300 bp were filtered. Assembly summary statistics were determined using QUAST (Gurevich et al., 2013) (version 2.3). The remaining contigs were identified as ***clean reads*** for the subsequent analyses.

### 2.3 Taxonomic and functional annotation

Open reading frames (ORFs) from each assembled contig were predicted using MetaGeneMark browser (Zhu et al., 2010) (http://exon.gatech.edu/meta_gmhmmp.cgi,version 3.26). Then, the predicted genes were clustered as representative non-redundant gene catalog using MMseqs2 (Steinegger and Söding, 2017) (version 12-113e3), and the thresholds of sequence identity and coverage were set as 95% and 90%, respectively. Protein sequences of the above-mentioned catalog were aligned to Non-Redundant Protein Database (Nr Database, NCBI) using DIAMOND (Buchfink et al., 2015) (version 0.9.29) for taxonomic annotations, during which the cutoff of expected value (E-value) was set as 10^-5^. For the subsequent analyses, taxonomic annotation on species level was used.

Additionally, multiple types of functional annotation were carried out by comparing the clean reads with specific databases with specific tools. Due to significant differences in environmental complexity between the two occupations being studied, several databases related to microbial gene expression, pathogenic bacteria, and antibiotics were primarily selected for functional annotation. These annotating databases included: **i)** annotation against the Kyoto Encyclopedia of Genes and Genome (KEGG) database (https://www.kegg.jp), performed with DIAMOND (Buchfink et al., 2015) (version 0.9.29, setting E-value cutoff as 10^-5^); **ii)** annotation against the Comprehensive Antibiotic Research Database (CARD, https://card.mcmaster.ca/), performed with the Resistance Gene Identifier tool (McArthur et al., 2013) (RGI, version 4.2.2) and provided by the database with default parameters; and **iii)** BLASTP search against the following three databases, performed with BLAST+ tool (Camacho et al., 2009) (version 2.2.31+, setting E-value cutoff as 10^-5^): set A of the virulence factor database (VFDB, http://www.mgc.ac.cn/VFs); Pathogen Host Interactions Database (PHI-base, http://www.phi-base.org/); and antibacterial biocide and metal resistance genes (BacMet) database (http://bacmet.biomedicine.gu.se/). Annotated functional genes were then clustered into several groups according to database-special rules, such as level 3 KEGG pathways. These groups are presented in detail in Table 3 and would be collectively referred to as “functional features” in the following analyses.

For the subsequent analyses, undetermined information, i.e., “unassigned” or “uncultured” species and reads cannot be functional annotated, was omitted, and the concept “relative abundance” means the proportion of the absolute abundance of the corresponding feature in all “determined” features. Based on the relative abundance data of the six types of annotation, primary comparison was carried out with the following process: i) The normality of relative abundance data for the two groups was respectively tested using the Shapiro-Wilk test; ii) The homogeneity of variance between two groups of data was tested using Levene's test; iii) For species that both of the two groups of data followed a normal distribution and have homogeneous variance, t-test was used to compare the means of the two groups. Otherwise, the Wilcoxon rank sum test was used to compare the median of the two groups.

### 2.4 Bioinformatics and statistical analysis

Previous research studies have shown that there are significant differences in the microbial composition between different body parts, even within the same individual. Therefore, all of the subsequent analyses are conducted separately among saliva samples or fecal samples, without cross-comparisons between the two sample types. Based on the taxonomic annotation (on species level) or the five types of functional annotation mentioned above, the composition information, including the absolute and relative abundance information, of the detected species or functional “features” in the samples can be obtained. Based on such information, multiple assessments of the sequencing data were conducted.

#### 2.4.1 Alpha diversity analysis

Within each sample, the Shannon index was calculated as an indicator of α diversity with Equation (1), where *m* denotes the total number of features detected in the sample and *p*_i_ represents the relative abundance of the *ith* taxon or functional feature. Then, for each annotation method and each sample type, five times of t-test or Wilcoxon rank sum test were conducted to observe whether there are significant differences in α diversity distribution between groups: (i) three times between the three sample groups (MLa, MSt, and FSt); (ii) between male smokers and male non-smokers; and (iii) between male drinkers and male non-drinkers. The choice of t-test or Wilcoxon rank sum test is made based on whether the Shannon indices of each group follow a normal distribution and have homogeneous variance, which is similar to section 2.3.


(1) $$\begin{array}{l} Shannon\;index=-\overset{m}{\underset{i=1}{\sum }}\left(p_{i}\log_{2}p_{i}\right) \end{array}$$

#### 2.4.2 Beta diversity analysis

Although the Shannon index can provide valuable insights into the diversity and evenness of features within individual samples, its calculation focuses solely on within-sample diversity and overlooks between-sample differences. Put simply, if there is no significant difference in the Shannon indices of two groups of samples, it does not necessarily mean that there are no differences in the distribution of microbial features between the two samples. To provide a more precise evaluation of the differences in distribution of specific features among different groups of samples, β diversity is calculated between different samples. Hellinger distance (HD) was calculated as an indicator of β diversity for each sample pair, with Equation (2), where *n* denotes the total number of features detected in the two sample and symbols “*a*_i_/*b*_i_” the relative abundance of the *ith* taxon or functional feature in the two samples, respectively. R package “*vegan*” version 2.6-4 (Oksanen et al., 2022) was used in the calculation.


(2) $$\begin{array}{l} HD=\sqrt{\overset{n}{\underset{i=1}{\sum }}\frac{{\left(\sqrt{a_{i}}-\sqrt{b_{i}}\right)}^{2}}{2}} \end{array}$$

Three types of principal coordinates analyses (Legendre and Legendre, 2012) (PCoA) were conducted per sample type per feature type, to explore the differences in feature composition among different groups: (i) that based on all the 100 samples divided into 6 groups; (ii) that based on 40 saliva male samples divided into complementary groups from two dimensions (smokers vs. non-smokers; drinkers vs. non-drinkers); (iii) that similar to ii) based on 40 fecal male samples. Additionally, 48 analysis of similarity (ANOSIM) tests were performed to evaluate whether the difference between groups was significantly distinguishable from the difference within groups. This encompassed four distinct categories per sample type ( × 2) per feature type ( × 6): **i)** between male students and female students; **ii)** between male laborers and male students, **iii)** between male drinkers and male non-drinkers; and **iv)** between male smokers and male non-smokers.

#### 2.4.3 LEfSe analysis based on taxonomic annotated information

The Linear Discriminant Analysis (LDA) Effect Size (LEfSe) method (Segata et al., 2011) is utilized to compare saliva or fecal samples collected from male students with those collected from male laborers. This approach integrates statistical tests with linear discriminant analysis to detect species that exhibit different abundance between the groups and offers both statistical significance and an estimation of effect size. A species is deemed differentially abundant between the two groups if the absolute value of the 10 logarithm of the species' LDA score exceeds 4.

### 2.5 Construction of models estimating the occupation of unknown samples

Twelve Random Forest (RF)-based models were performed to estimate the occupation of unknown samples for each sample type and feature type, based on the taxa or functional annotation information of 80 male samples. This was accomplished using the R package “*randomForest*” version 4.7.1 (Liaw and M., 2002). For the construction of each model, features that were not detected in at least 10 samples were filtered out, and the count information on the remaining features was transformed and normalized using a Bayesian-multiplicative (BM) treatment (Martín-Fernández et al., 2015) with the R package “*zCompositions*” version 1.5 (Palarea-Albaladejo and Martín-Fernández, 2015). In this treatment, the 0 data were replaced by its posterior Bayesian estimate, and the non-zero counts were proportionally reduced to ensure that the sum of all feature output values for each sample is 1.

A recursive feature elimination (RFE) process was applied as follows: **i)** 100 random forests were generated based on all taxa or functional features, during which the variable importance parameter "mean decrease accuracy" was calculated. The taxa or functional features were then ranked according to the average of this parameter. **ii)** The feature with the lowest importance value was omitted, and another 100 random forests were generated with the same setting as in step i). **iii)** Step ii) was repeated until there was only one feature left, and the “best” model, i.e., the model with the minimum feature number, achieving the highest accuracy in the two sample sets, was output. The overall process of the model is shown in Algorithm 1. The 12 models (2 sample types × 6 feature types) were then compared with each other. The R code (version 4.3.1) for the RFE process is presented in Supplementary File S1 in Data Sheet 2, and the count data for the code is presented Supplementary File S2 in Data Sheet 3. It should be noted that samples collected from female students, such as the “FStF” and “FStS” groups, were omitted in the model construction to exclude the influence of gender, as shown in the Discussion section.

**Algorithm 1:**
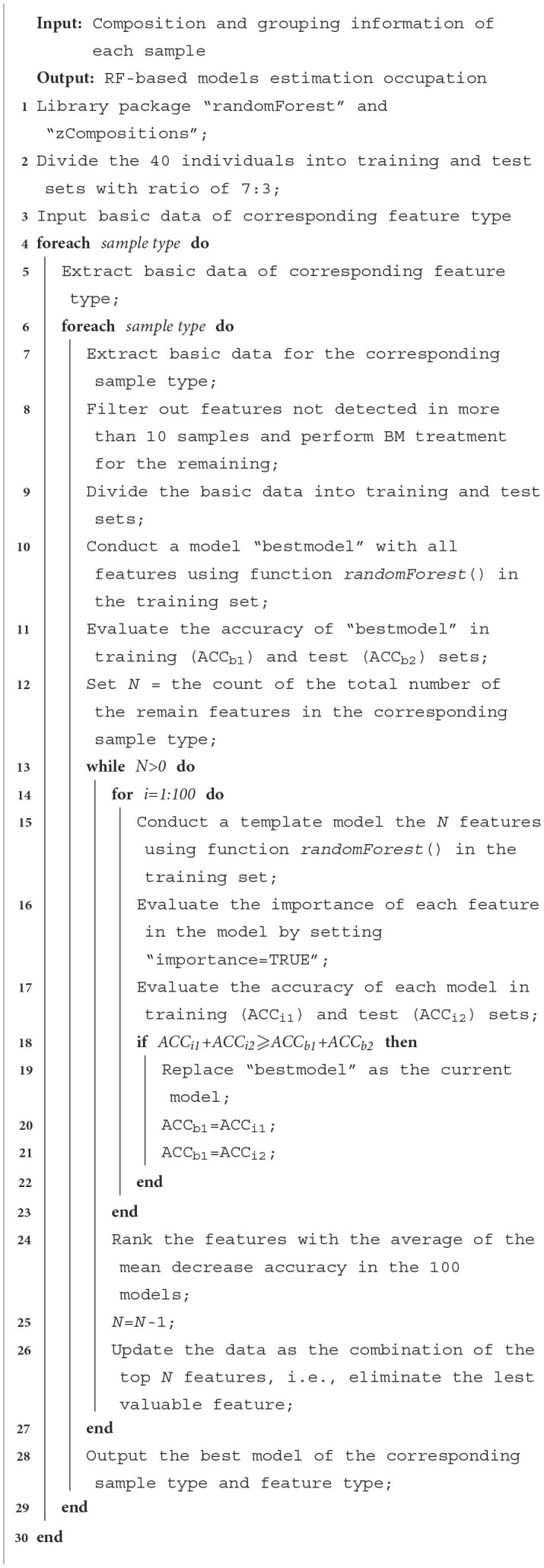
Conduction of Random Forest-based models.

## 3 Results

A total of 6.75 × 10^11^ base pairs of *clean data* were identified after quality control using Trimmomatic, of which 2.28 × 10^11^ base pairs (33.73%) were matched as human genome. Consequently, 362,588,258 non-host *clean reads* were assembled using MEGAHIT across all 100 samples, with 79,816,837 and 282,771,421 in saliva and fecal samples, respectively. Taxonomic and functional annotations were performed based on these *clean reads*.

### 3.1 Annotation information

#### 3.1.1 Taxonomic annotation information

**Figure 1:**
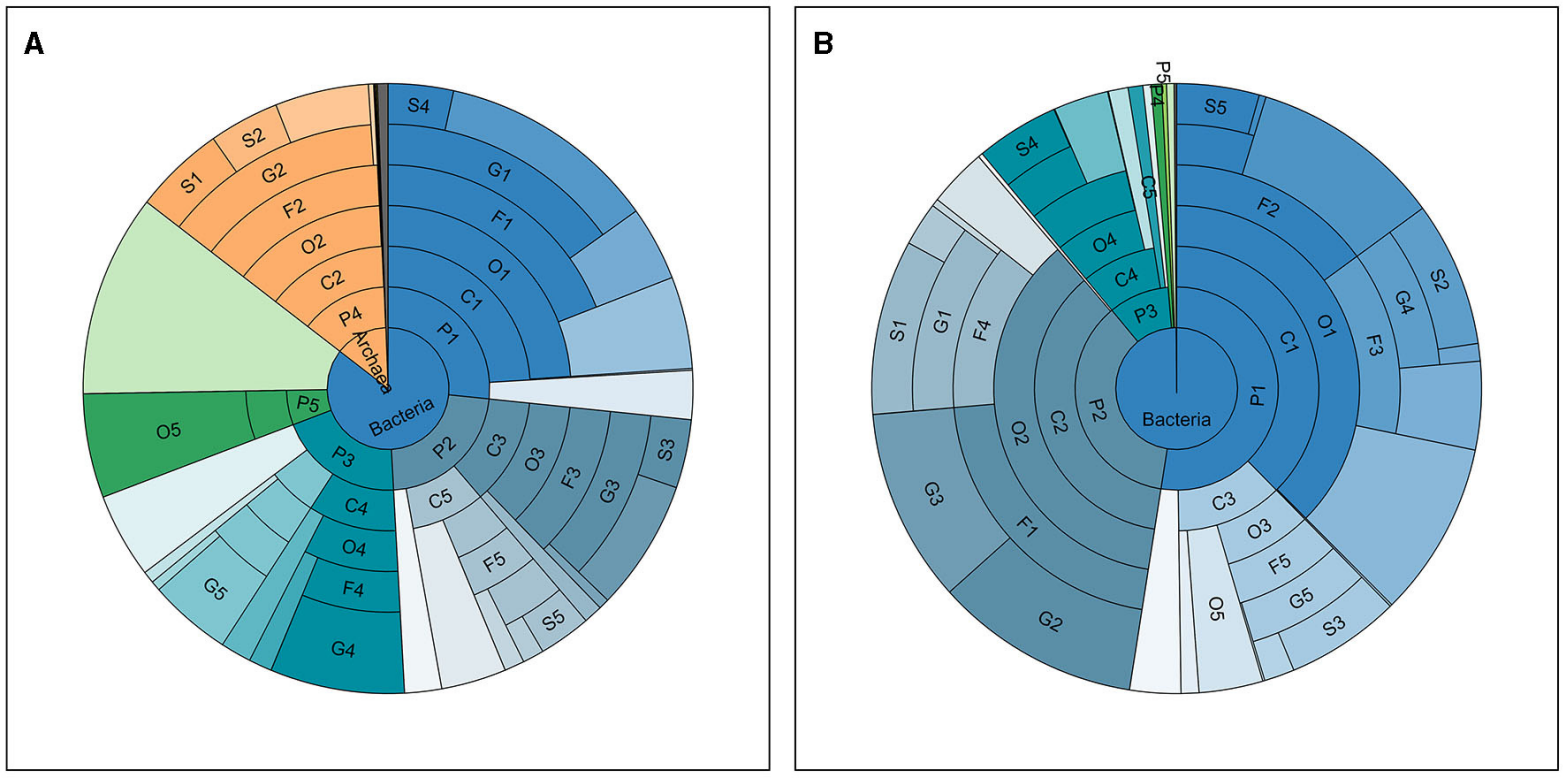
Taxonomic distribution in all samples. The abundance distribution of taxa detected and annotated in all saliva **(A)** and fecal **(B)** samples, at seven taxonomic levels, namely, from inside out, Kingdom (K), Phylum (P), Class (C), Order (O), Family (F), Genus (G), and Species (S). The ranks of top taxa are labeled with numbers after the abbreviations of the level, the names and proportions of which are shown in Table 2.

Taxonomic annotation was carried out at seven levels, namely, Kingdom, Phylum, Class, Order, Family, Genus, and Species. Supplementary Table S1 in Data Sheet 1 shows the number of taxa annotated and classified in each sample at each of these levels and the total number of annotated taxa in all samples. A total of ~17,000 species (with 243,325,739 reads, 67.11% in all clean reads) were annotated in all samples. Meanwhile, an average of ~2,400 species were annotated for each sample and the phenomenon where the average number of detected taxa per sample was much smaller than the total number in all samples showed in every taxonomic level lower than kingdom, meaning that the vast majority of the taxa were detected in limited number of samples. For instance, *Clostridium* sp. IBUN62F, an unclassified species in the genus *Clostridium*, was detected in only 1 sample (MSt17F) with 902 reads. Top taxa at each level in the two types of samples are presented in Table 2, and the abundance distribution of these taxa in the two types of samples is shown in Figure 1. It can be observed that there is an obvious difference between the top taxa of the of the two sample types. For instance, the *Bacteria* kingdom accounted for over 99.5% of fecal samples, while the *Archaea* kingdom occupied ~15% of reads in saliva samples. On each lavel, the proportion of the dominant taxa in fecal samples was higher than the saliva ones.

**Table 2:** Top taxa annotated in the two types of samples.

		**Saliva samples**		**Fecal samples**	
**Level**	**Rank**	**Taxon name**	**Proportion**	**Taxon name**	**Proportion**
Kingdom	1	*Bacteria*	85.47%	*Bacteria*	99.86%
	2	*Archaea*	13.96%	*Viruses*	0.12%
	3	*Viruses*	0.39%	*Archaea*	0.02%
Phylum	1	*Bacteroidota*	26.63%	*Bacillota*	52.48%
	2	*Proteobacteria*	22.50%	*Bacteroidota*	36.51%
	3	*Bacillota*	20.07%	*Proteobacteria*	9.67%
	4	*Euryarchaeota*	13.90%	*Actinomycetota*	0.56%
	5	*Fusobacteriota*	5.52%	*Chlamydiota*	0.26%
Class	1	*Bacteroidia*	24.04%	*Clostridia*	37.65%
	2	*Thermococci*	13.84%	*Bacteroidia*	36.27%
	3	*Betaproteobacteria*	12.13%	*Negativicutes*	12.12%
	4	*Bacilli*	10%	*Gammaproteobacteria*	8.45%
	5	*Gammaproteobacteria*	8.42%	*Betaproteobacteria*	0.79%
Order	1	*Bacteroidales*	23.94%	*Eubacteriales*	37.53%
	2	*Thermococcales*	13.78%	*Bacteroidales*	36.27%
	3	*Neisseriales*	11.13%	*Selenomonadales*	7.79%
	4	*Lactobacillales*	8.36%	*Enterobacterales*	7.41%
	5	*Fusobacteriotales*	5.52%	*Veillonellales*	3.39%
Family	1	*Prevotellaceae*	19.07%	*Bacteroidaceae*	21.17%
	2	*Thermococcaceae*	13.77%	*Lachnospiraceae*	14.93%
	3	*Neisseriaceae*	11.13%	*Oscillospiraceae*	13.30%
	4	*Streptococcaceae*	7.13%	*Prevotellaceae*	11.99%
	5	*Pasteurellaceae*	4.96%	*Selenomonadaceae*	7.79%
Genus	1	*Prevotella*	15.11%	*Prevotella*	11.59%
	2	*Thermococcus*	13.50%	*Phocaeicola*	10.90%
	3	*Neisseria*	10.57%	*Bacteroides*	10.26%
	4	*Streptococcus*	7.13%	*Faecalibacterium*	8.63%
	5	*Veillonella*	4.41%	*Megamonas*	7.68%
Species	1	*Thermococcus nautili*	4.81%	*P. copri*	9.27%
	2	*Thermococcus henrietii*	3.72%	*Faecalibacterium prausnitzii*	7.70%
	3	*Neisseria flavescens*	3.63%	*Megamonas funiformis*	6.07%
	4	*Prevotella melaninogenica*	3.46%	*E. coli*	4.41%
	5	*Haemophilus parainfluenzae*	2.77%	[Eubacterium] *rectale*	4.37%

**Figure 2:**
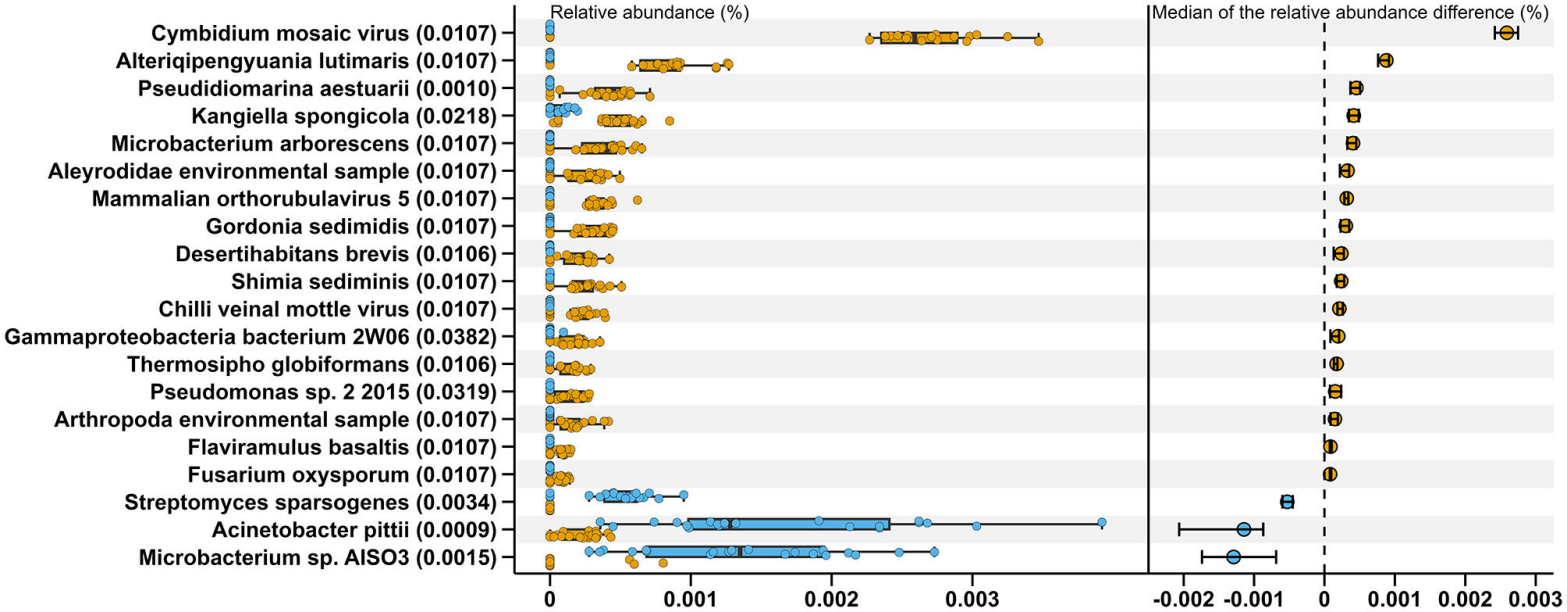
Twenty species exhibiting significant differences in relative abundance between MStF (blue) and MLaF (Orange) samples. The boxplots on the left represent the relative abundance of these species in each sample, from which 400 (20×20) differences between the two types of samples were calculated for each species. The right section displays the medians of these differences, along with their 95% confidence intervals. The adjusted *P*-values, resulting from the Wilcoxon rank sum tests conducted between the two groups, are presented in the parentheses on the y-axis.

Comparisons between MSt and MLa samples were carried out based on the distribution of relative abundance on each species annotated in the two types of samples with process mentioned in Section 2.3. There were nine and three species met the criteria for conducting **t**-tests for saliva and fecal samples, respectively, all of which produced adjusted *P*-value of 0.05. Within the other species, there was one showed significant difference after Bonferroni correction (adjusted *P*-value=0.045) between saliva samples of the two male groups, namely, *Lachnoclostridium edouardi*. Meanwhile, for fecal samples, 20 showed significant differences in relative abundance between the two groups after the Wilcoxon rank sum test (see Figure 2).

#### 3.1.2 Functional annotation information

Five types of functional annotation methods were applied, and the number of annotated functional genes and the proportion of corresponding reads in the total reads are presented in Table 3. Similar to the taxonomic annotation results, t-tests or Wilcoxon rank sum tests were carried out based on the relative abundance of functional features between MSt and MLa samples, per sample type per database, and significant difference was found in 14 features after Bonferroni correction (adjusted *P*-value < 0.05, see Table 3).

**Table 3:** Functional annotation information.

			**Annotated**		**Significant features**		
**Database**	**Feature name**	**Type**	N^*^	Proportion^**^	Method^***^	**Feature name**	***P*-value^†^**
KEGG	KEGG pathway level 3	Saliva	166	58.27%	w	ko03420	0.0333
			w	ko03430	0.0115
			t	ko00240	0.0296
		Fecal	161	70.12%	w	ko03013	0.0011
			w	ko04141	0.0344
CARD	Antibiotic resistance type	Saliva	27	2.58%	-^‡^	-	-
		Fecal	27	4.58%	-	-	-
VFDB	Virulence Factors	Saliva	291	3.90%	w	VF0126	0.0049
			w	VF0191	0.0354
			w	VF0277	0.0311
		Fecal	327	5.18%	-	-	-
PHI-base	PHI-ID	Saliva	2188	10.16%	w	PHI:128/PHI:6818	0.0048
			w	PHI:6370	0.0054
		Fecal	2145	13.60%	w	PHI:6612	0.0024
			w	PHI:7677	0.0336
			w	PHI:7679	0.0393
BacMet	BacMet gene name	Saliva	474	4.00%	w	cdeA	0.0075
		Fecal	520	3.41%	-	-	-

^*^the number of annotated features; ^**^the proportion of the annotated reads in the total detected clean reads, i.e., 79,816,837 in saliva samples and 282,771,421 in fecal ones; ^***^the method used in the comparison: w, Wilcoxon rank-sum test; t, t-test; ^†^The *P*-values are adjusted through Bonferroni correction; ^‡^Symbol “-” indicates that no significant difference was found in all features annotated.

### 3.2 Diversity analysis between different groups

#### 3.2.1 Alpha diversity analysis

**Figure 3:**
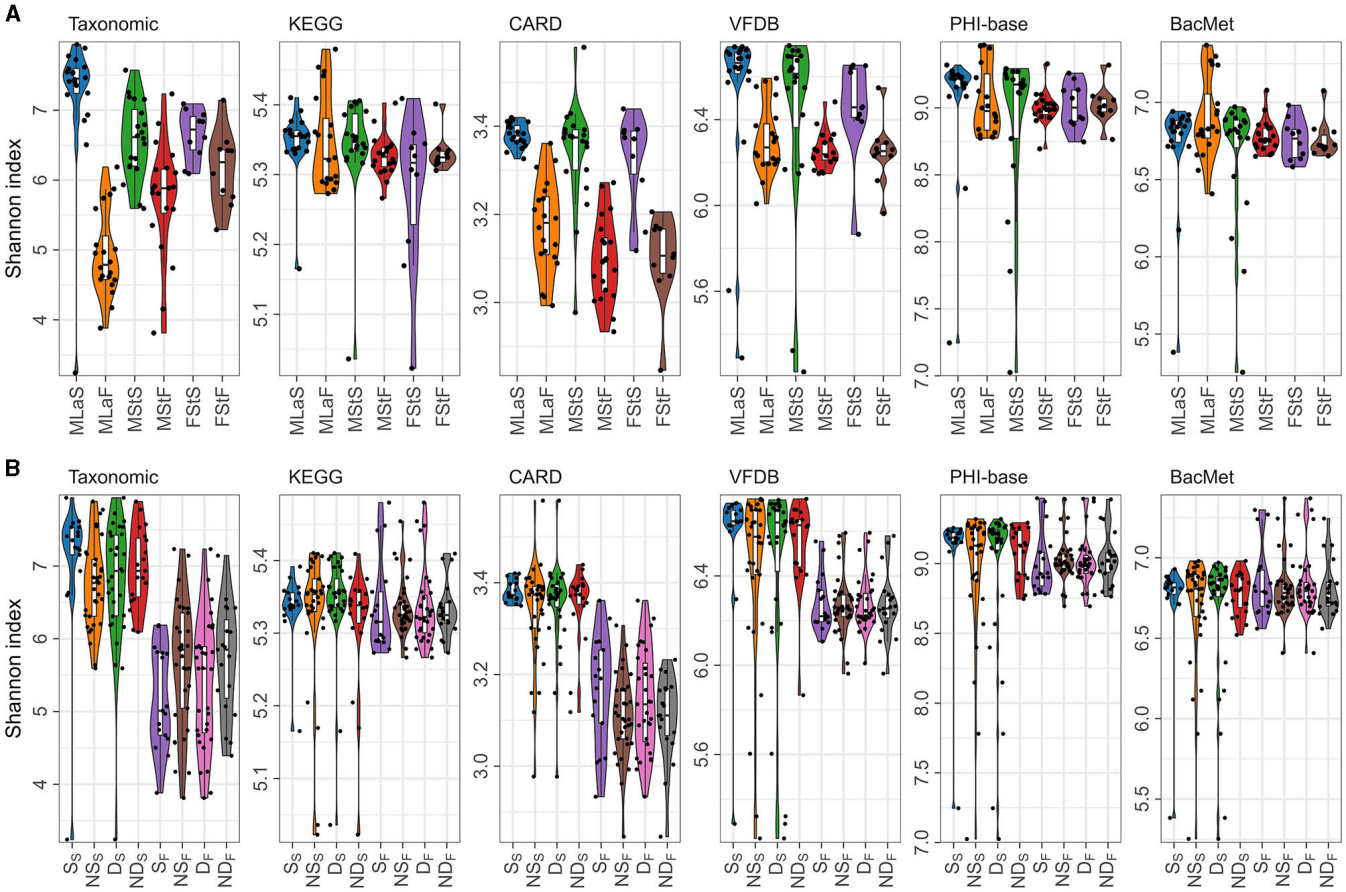
Shannon indices calculated based on different annotation methods. **(A)** Distribution of Shannon indices in different sample groups; **(B)** The distribution of Shannon indices in male samples, grouped by lifestyle habits and sample types, denoted by letters with subscripts. In the subscript, “S” represents saliva samples and “F” represents fecal samples, while the letters in normal font represent the specific habits: “S” for smokers, “NS” for non-smokers, “D” for drinkers, and “ND” for non-drinkers.

Based on the relative abundance information achieved in the above section, Shannon index was calculated per sample per annotation method, as shown in Figure 3A. Additionally, the male samples were divided into opposite groups based on whether they smoke or drink alcohol, as shown in Figure 3B. As mentioned in Section 2.4.1, 30 times of t-tests or Wilcoxon rank-sum tests were performed, and the results are shown in Table 4. Regardless of the annotation method used, the Shannon index distribution of MSt samples did not differ significantly from that of FSt samples of the same type. Meanwhile, significant differences were found between MSt samples with MLa samples based on taxonomic annotation information, which roughly suggested the possible potential of taxonomic information in the distinguishing of students from labouers. Other comparisons did not reveal significant differences, apart from the contrast between smokers and non-smokers, based on species annotation information in saliva samples.

**Table 4:** Functional annotation information.

**Annotation**	**Sample**	**MLa**	**FSt**	**MLa**	**Male smoker**	**Male drinker**
**Method**	**Type** ^*^	**MSt**	**MSt**	**FSt**	**Male non-smoker**	**Male non-drinker**
Taxonomic	Saliva	**0.0001(w)** ^**^	1(t)	**0.0010(w)**	**0.0480(w)**	0.0594(w)
	Fecal	**0.0038(t)**	0.3238(t)	**5.0210** **×10**^**(−5)**^	0.2825(t)	0.5238(t)
KEGG	Saliva	1(w)	0.2234(w)	0.0933(w)	0.0751(w)	0.6103(w)
	Fecal	1(w)	1(w)	1(w)	0.3855(w)	0.9240(w)
CARD	Saliva	1(w)	1(t)	0.9845(w)	0.4161(w)	0.4248(w)
	Fecal	0.0613(w)	1(w)	0.2019(w)	0.1503(t)	0.4668(t)
VFDB	Saliva	0.4960(w)	1(w)	0.0828(w)	0.3423(w)	0.4828(w)
	Fecal	1(t)	1(t)	0.9739(w)	0.7049(w)	0.8736(w)
PHI-base	Saliva	1(w)	1(w)	0.0647(w)	0.7868(w)	0.3709(w)
	Fecal	1(w)	1(w)	1(w)	0.1647(w)	0.8486(w)
BacMet	Saliva	1(w)	1(w)	0.7453(w)	0.4319(w)	0.8238(w)
	Fecal	0.3242(w)	0.8595(w)	0.2019(w)	0.7049(w)	0.6557(w)

^*^For each annotation method and each sample type, five types of comparison were performed, the two groups are presented in the table header; ^**^Adjusted *P*-values for each comparison are listed with the comparing method labeled in the following parentheses: w, Wilcoxon rank-sum test; t, t-test; Comparisons with *P*-value < 0.05 are labeled red.

#### 3.2.2 Beta diversity analysis

**Figure 4:**
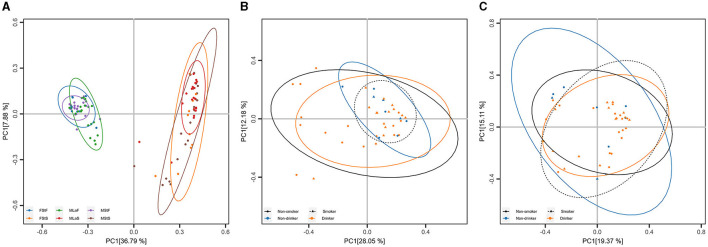
Principal Coordinate Analysis (PCoA) based on taxonomic annotation. **(A)** PCoA results based on Hellinger distance calculated within all samples, each point represents a sample, and the group is denoted by its color. **(B)** PCoA results based on Hellinger distance calculated within male saliva samples, each point represents a sample, and the living habits are indicated by its shape (smoking) and color (drinking). **(C)** Results similar to **(B)** within male fecal samples.

PCoA and ANOSIM were carried out based on Hellinger distance (HD) calculated after each of the six annotation methods. The results achieved based on taxonomic annotation are presented as examples in Figure 4, showing that the fecal samples can be distinguished from saliva samples, while samples of the same type can hardly be separated, considering or not considering lifestyle habits including drinking and smoking. Similar results can be observed through PCoA based on information achieved by the other five types of annotation methods, as shown in Supplementary Figure S1 in Data Sheet 1 and Image 1. For each annotation method, eight times of ANOSIM were performed, and the details of each analysis, as well as the results, are shown in Table 5. It can be observed that for all annotation methods, if comparing students of different genders, the inter-group differences were not significantly higher than the corresponding inner-group differences (*P*-value>0.05), indicating that the compositions of microbiota between male and female students are similar to each other. Similar results were observed when comparing male drinkers with non-drinkers or comparing male smokers with non-smokers. On the other hand, the situation was exactly the opposite when comparing samples collected from male students with those from male laborers, where the inter-group differences significantly exceeded the corresponding inner-group ones. This indicates a certain degree of difference in microbial composition between individuals engaged in these two types of work, which also suggests the feasibility of further screening high-quality taxa or features to accurately distinguish between the two groups of individuals.

**Table 5:** Results of ANOSIM.

**Comparison**								
**Individual range**	**Groups compared**	**Sample type**	**Method**	**R**	***P*** **-value** ^*^	**Method**	**R**	***P*** **-value**
within students	Males vs. Females	Saliva samples	Taxonomic	-0.0451	0.666	VFDB	-0.0258	0.554
			KEGG	0.1030	0.146	PHI-base	-0.0262	0.575
			CARD	-0.0197	0.542	BacMet	-0.0329	0.580
		Fecal samples	Taxonomic	-0.0016	0.440	VFDB	-0.0465	0.640
			KEGG	-0.0053	0.472	PHI-base	-0.0526	0.695
			CARD	-0.0471	0.711	BacMet	-0.0525	0.725
within males	Laborers vs. students	Saliva samples	Taxonomic	0.1323	**0.003**	VFDB	0.0774	**0.002**
			KEGG	0.0850	**< 0.001**	PHI-base	0.0879	**0.004**
			CARD	0.0658	**0.005**	BacMet	0.1008	**< 0.001**
		Fecal samples	Taxonomic	0.0810	**0.032**	VFDB	0.1256	**0.002**
			KEGG	0.0918	**0.011**	PHI-base	0.1323	**0.002**
			CARD	0.0734	**0.031**	BacMet	0.1336	**0.004**
	Drinkers vs. non-drinkers	Saliva samples	Taxonomic	-0.1291	0.900	VFDB	-0.1795	0.958
			KEGG	-0.1565	0.903	PHI-base	-0.1839	0.948
			CARD	-0.1625	0.923	BacMet	-0.1733	0.936
		Fecal samples	Taxonomic	-0.0496	0.686	VFDB	-0.0459	0.641
			KEGG	-0.0768	0.752	PHI-base	-0.0590	0.710
			CARD	-0.0719	0.772	BacMet	-0.0348	0.567
	Smokers vs. non-smokers	Saliva samples	Taxonomic	0.0512	0.097	VFDB	0.0047	0.378
			KEGG	0.0130	0.296	PHI-base	0.0164	0.268
			CARD	0.0061	0.361	BacMet	0.0194	0.245
		Fecal samples	Taxonomic	0.0008	0.440	VFDB	0.0671	0.057
			KEGG	0.0818	**0.037**	PHI-base	0.0812	0.053
			CARD	0.0497	0.115	BacMet	0.0726	0.060

^*^The *P*-value in ANOSIM is obtained by comparing the observed test statistic (R) with R distribution generated by random permutations of the original data. The number of permutations is set as 999 in our research, meaning that the precision of *P*-value is 0.001, and the symbol “ < 0.001” means that none of the 999 permutations achieved R greater than the observed one. Comparisons with a *P*-value < 0.05 are indicated in red bold font.

### 3.3 LEfSe analyses based on species annotation

**Figure 5:**
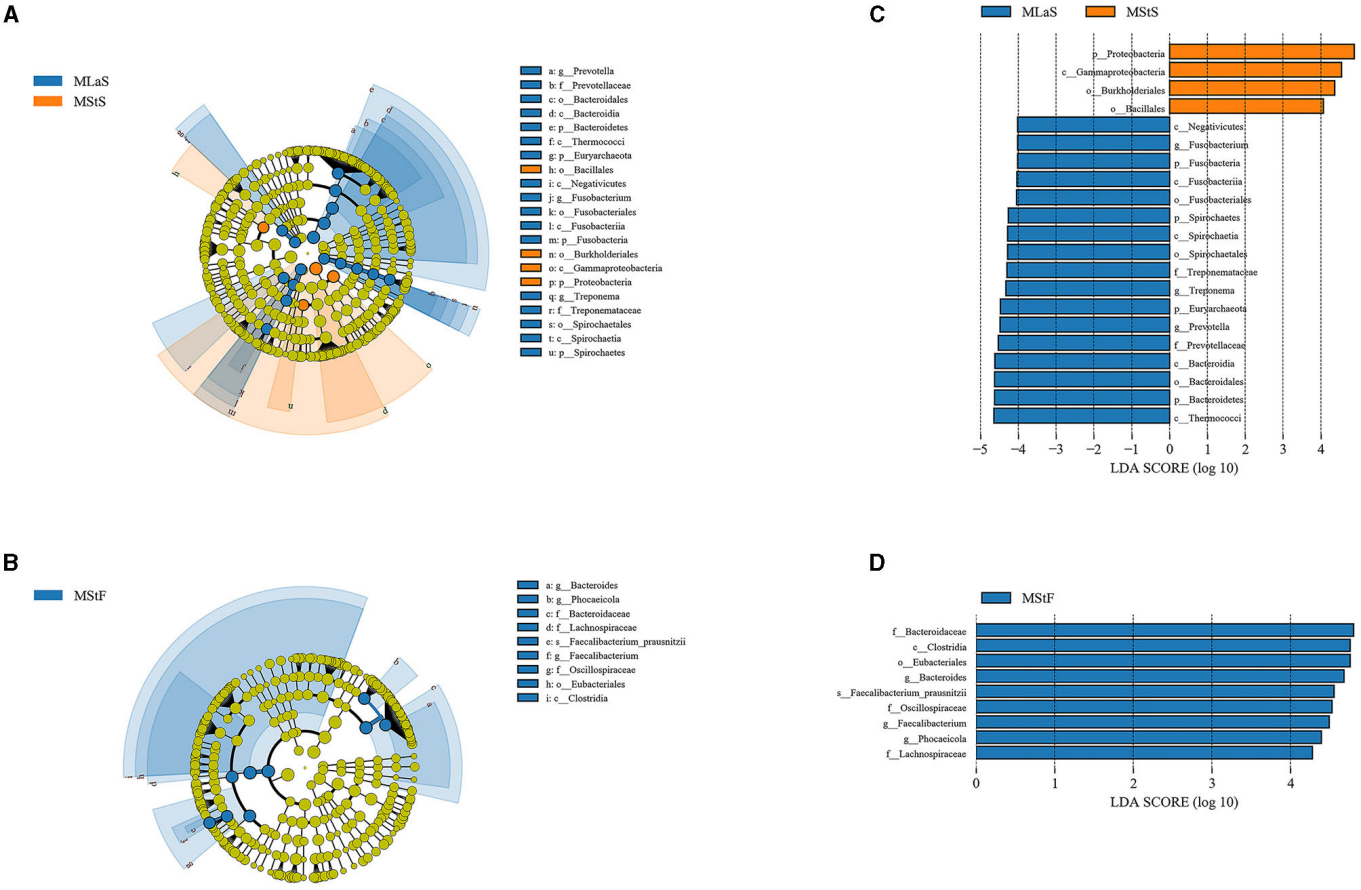
Results from LEfSe analyses comparing the MLa and MSt groups. **(A, B)** Depict the taxonomic branching diagram in saliva **(A)** and fecal **(B)** samples, with taxa having an absolute value log_10_LDA score greater than 4 labeled. Taxa are colored blue if they are more abundant in MLa samples or orange if they are more abundant in MSt samples. **(C, D)** Show the log_10_LDA score of differentially abundant taxa in saliva **(C)** and fecal **(D)** samples.

LEfSe analyses were performed based on the relative abundance data of saliva or fecal samples between MLa and MSt groups. As shown in Figure 5, 21 and 9 taxa are differentially abundant between the two groups in saliva and fecal samples, respectively, using the evaluation criteria shown in Section 2.4.3.

### 3.4 Construction of models distinguishing students with laborers based on species and functional annotation

All models were constructed and verified with the same division of training and test sets, which are presented in Supplementary Table S2 in Data Sheet 1.

#### 3.4.1 Models based on taxonomic annotation at species level

For saliva samples, 615 species were detected in at least 30 samples and used in the model construction. After 100 times of RF process using all the species (i.e., the first round of while loop in line 1-1 of Algorithm 1), a model was constructed with 100% accuracy in both training and test sets. RFE process was then carried out, resulting in a model based on three species with the same 100% accuracy. Species used in the model are presented in Supplementary Table S3-1 in Data Sheet 1, and the model is provided as “File_S3_1.Rdata” in Data Sheet 4.

For fecal samples, on the other hand, 1350 species were detected in 30 or more samples, which is much higher than the saliva samples. However, after the first round of while loop in Algorithm 1, the accuracy of the best model using all the 1,350 species in test set was 83.33%. After RFE process, the best model was achieved when the number of applied species decreased to 5, the accuracy of which in both training and test sets was 100%. Species used in the model are presented in Supplementary Table S3-1 in Data Sheet 1, and the model is provided as “File_S3_2.Rdata” in Data Sheet 4.

#### 3.4.2 Models based on functional annotation

Similar construction process was applied based on data achieved by functional annotation using 5 databases as mentioned above, resulting in 10 RF models, and the details of such 10 models are presented in Table 6, and features selected in each model are presented in Supplementary Table S3 in Data Sheet 1. Pathway annotation data achieved from the KEGG database using fecal samples can provide the best accuracy in training and test sets with the least number of features. In such a model, a single pathway is included, i.e., “ko04145” or Phagosome, the cellular process that involves engulfing and degrading foreign particles, such as bacteria and viruses.

**Table 6:** RF models constructed based on taxonomic and functional annotation.

**Annotation**		**All feature in** ≥30 **samples**			**Best model**			**Output information**	
**Method**	**Type**	***N***	**ACC_b1_**	**ACC_b2_**	***N***	**ACC_b1_**	**ACC_b2_**	**Species**	Model^*^
Taxonomic	Saliva	615	100%	100%	3	100%	100%	Supplementary Table S3-1	_1
	Fecal	1,350	100%	83.33%	5	100%	100%	Supplementary Table S3-1	_2
KEGG	Saliva	120	100%	91.67%	19	100%	100%	Supplementary Table S3-1	_3
	Fecal	129	100%	91.67%	1	100%	100%	Supplementary Table S3-1	_4
CARD	Saliva	23	100%	83.33%	18	100%	91.67%	Supplementary Table S3-1	_5
	Fecal	25	100%	83.33%	8	100%	91.67%	Supplementary Table S3-1	_6
VFDB	Saliva	179	100%	91.67%	17	100%	91.67%	Supplementary Table S3-2	_7
	Fecal	195	100%	75%	7	100%	91.67%	Supplementary Table S3-2	_8
PHI-base	Saliva	943	100%	91.67%	11	100%	91.67%	Supplementary Table S3-2	_9
	Fecal	1,139	100%	83.33%	3	100%	83.33%	Supplementary Table S3-2	_10
BacMet	Saliva	255	100%	91.67%	7	100%	91.67%	Supplementary Table S3-2	_11
	Fecal	337	100%	75%	6	100%	83.33%	Supplementary Table S3-2	_12

^*^Best models are provided as 12 .Rdata files named as “File_S3_X.Rdata” in Data Sheet 4.

## 4 Discussion

In this investigation, we utilized metagenomic sequencing data to compare and evaluate the variation in the composition of saliva and fecal microbiota among different groups based on multiple perspectives, including gender, occupation, and smoking or drinking habits. Our findings indicate that there is no significant difference in the oral and gut microorganisms among student groups considering the two genders, regardless of the type of annotation (taxonomic or functional) or the analysis methods used (α diversity, PCoA, or ANOSIM). However, we observed that smoking and drinking habits can lead to distinguishable variations in the distribution of oral and gut microbiota, which can be detected using some of the methods employed in this study. Additionally, we found that within the male samples, the two occupations we focused on, students and laborers, can be differentiated based on information about their oral or gut microbiota, using each of the aforementioned methods. To further analyze this distinction, we used recursive feature elimination processes with random forest classifiers and successfully developed 12 models that accurately distinguish individuals with the two occupations, the best accuracy of which was 100% in both training and test sets. Our findings provides a preliminary support of the potential application value of microbial information in occupation or living habit discrimination and estimation.

As mentioned in the Introduction section, compared with traditional amplicon-based microbial sequencing methods, the metagenomic sequencing method applied in this study can reflect the distribution of microorganisms, hosts, and environmental factors in specific samples from a more comprehensive and macroscopic perspective. For instance, the *Archaea* kingdom occupied ~15% in all annotated reads of the saliva samples and the two species *Thermococcus nautili* and *Thermococcus henrietii* of genus *Thermococcus* ranked the top two species in those samples, which cannot be revealed by traditional 16S rRNA sequencing without extra design of primers (Chaudhari et al., 2020; de Andrade et al., 2020). Within the *Bacteria* kingdom, the top taxa are nearly identical to previous studies (Chaudhari et al., 2020; de Andrade et al., 2020; Ma et al., 2021). Throughout the study, instead of analyzing the microbial information directly based on the reads data, we considered the microbiome information as compositional and employed multiple methods considering the relative abundance information of the annotated sequences. These methods include the Shannon index, Hellinger distance, the LEfSe analysis, and the BM treatment during the model construction process. This approach is taken due to the nature of sequencing instruments, which can deliver reads only up to the capacity of the instrument. Therefore, the sequencing results represent a fixed-size, random sample of the relative abundance of the molecules in the underlying ecosystem, and the number of reads obtained is irrelevant, as discussed in Gloor et al. (2017).

A variety of factors shaped the personal specificity and stability of oral and fecal microbiota community in adults (Martino et al., 2022), including gender, age, genetic background, living environment, health status, medicine contraction, and living habits such as drinking and smoking. When constructing occupation distinguishing models, the influence of gender is excluded by eliminating female student samples due to the absence of samples collected from female labouers. Although there was no significant difference found between the MSt and FSt samples within the same sample type, regardless of the annotation method or diversity type (α or β) compared, it is not sufficient to conclude that the correlation between occupation and microbial information is unaffected by gender. It has been proven by multiple studies showing that the microbial composition differed between the two genders (Richardson et al., 2019; Nearing et al., 2020). Most students come from the same department and live in the same campus for a long time, resulting in similar eating habits and living rhythm. Studies have shown that the transmission of the oral microbiome occurred largely horizontally and is enhanced by the duration of cohabitation. There was a significant amount of strain sharing among individuals living together, with median strain sharing rates of 12% and 32% for the gut and oral microbiomes, respectively. And the time since cohabitation is positively correlated with strain sharing in the oral microbial community (Valles-Colomer et al., 2023). Meanwhile, the differences between smokers/drinkers and non-smokers/non-drinkers were omitted, based on the fact that significant difference between different genders/habits was not found in almost all related tests (see Figure 3, Table 5). However, it is found in multiple studies (Capurso and Lahner, 2017; Lin et al., 2020; Jia et al., 2021; Liao et al., 2022; Yu et al., 2024) that all these factors can affect the structure of oral and gut microbiota. For instance, Yu et al. (2024) analyzed saliva microbial information of 43 Korean participants through 16S rRNA sequencing, finding that specific microorganisms would distribute differently between individuals with different smoking or drinking habits. The difference between our results may be caused by the relatively small sample size in our study and the difference between sequencing methods and health conditions of the samples.

**Figure 6:**
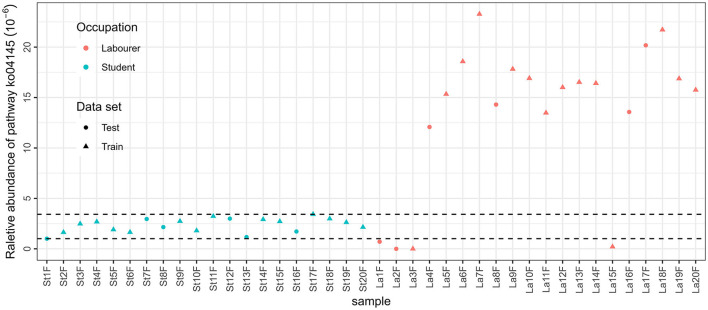
Relative abundance of pathway ko04145 in MStF and MLaF samples.

The results of ANOSIM showed that there were significant differences between male students and male laborers in saliva and fecal samples, no matter which annotation method is used. This provides theoretical support for the subsequent construction of classification models. Occupation is related to multiple of the aforementioned factors, affecting microbiota community, and the variations in occupations can be attributed to the discrepancies in the internal and external environments of microbial communities that are influenced by these factors. For example, the best model based on functional annotation method (KEGG database with fecal samples) included only one feature, which is related to the innate immune system. A closer look was taken to the relative abundance of the pathway in the total reads annotated in the KEGG database, as presented in Figure 6. The concentration of the relative abundance of this pathway in the samples from students was found to be limited to a narrow range. However, in the case of laborers, the relative abundance was distributed differently. Specifically, it was significantly higher compared with the students in the majority of samples (16 out of 20) but very low or even undetectable in the other four samples. This suggests that the laborers may be exposed to a more complex environment. Similarly, the three species involved in the taxonomic-saliva model, *[Eubacterium] brachy, Acinetobacter baumannii*, and *[Eubacterium] infirmum*, are usually related to oral diseases such as periodontitis or other health issues if detected in saliva (HOLDEMAN et al., 1980; CHEESEMAN et al., 1996; Peleg et al., 2008); the difference between their distribution in samples of the two occupations may also suggest the difference in environmental complexity between two occupational individuals and the resulting differences in health status.

In the construction of distinguishing models, we incorporated a preliminary step of eliminating features that were not observed in more than 25% of the tested samples. This step was implemented before the RF processes, with the aim of identifying more robust and universally applicable features for differentiating or predicting occupations.Nevertheless, this action may have rendered specific potentially valuable features ineffective in advance. For instance, 20 species showed significant difference between MStF and MLaF samples after the Wilcoxon test, as shown in Figure 2, within which the species *Streptomyces sparsogenes*was detected in 17 MStF samples but not identified in any fecal samples from laborers, while 14 other species (*Flaviramulus basaltis, Pseudidiomarina aestuarii, Alteriqipengyuania lutimaris, Shimia sediminis, Gordonia sedimidis, Microbacterium arborescens, Desertihabitans brevis, Thermosipho globiformans, Cymbidium mosaic virus, Mammalian orthorubulavirus 5, Chilli veinal mottle virus, Fusarium oxysporum, Aleyrodidae* environmental sample, *Arthropoda* environmental sample, and *Pseudomonas* sp. 2 2015) were not present in any MStF samples and were detected in at least 15 MLsF samples. However, all of these species occupied only ~0.0025% in the total abundance of fecal samples.

When applying taxonomic annotated information, both models based on saliva and fecal samples can offer 100% discriminative power, while the saliva model is formed by less features. However, the situation is reversed when constructing functional-annotation-based models. The reason for this may be that oral microbiota has been found to be more closely affected by the impact of external factors such as dietary, compared with gut microbiota (Valles-Colomer et al., 2023). Thus, the microbial information in saliva can be a better reflection of the environmental microbial community, resulting in a more diverse taxonomic distribution, as shown in Figure 1, and a better probability of showing difference between the two occupations is based on the taxonomic information as shown in Figure 3. After the selective effect of the digestive tract environment of the host on microorganisms, we observed a higher concentration of microbiota in the fecal samples. The dominant taxa were more abundant, while the species that showed significant differences between the two groups were less prevalent, as mentioned earlier. As a result, the overall difference of taxa composition between MStF and MLaF samples was less than that between MStS and MLaS samples. Functional factors, such as various reactions activated by the host due to microbial entry, as well as the way the responds of microbiota to the environment, have a significant impact on the composition of the gut microbiome. This means that there is a greater likelihood of observing functional annotated genes in fecal samples, finding distinct features.

When working with microbial data that have an extremely high number of features compared with the number of samples, we often encounter challenges related to overfitting (i.e., the problem when a model learns the training data too well and fails to generalize well to unseen data) and the “curse of dimensionality” (i.e., the phenomenon where the sample complexity of a learning algorithm increases exponentially with the number of features in the dataset). This emphasizes the importance of employing feature elimination as a solution. Recursive feature elimination (RFE) is a popular method for reducing the dimension of datasets while retaining relevant features. It recursively removes the least important features until a desired feature subset size is reached. RFE algorithm iteratively selects a subset of features and then measures their importance using a monomer classifier. The least important features are recursively removed from the dataset until a desired feature subset size is reached. By recursively removing the least important features, RFE can significantly reduce the dimensionality of the dataset while retaining relevant information for the target variable. This can improve the performance and computational efficiency of machine learning algorithms, reduce overfitting, and improve generalization accuracy. Random forest (RF) is selected as the monomer classifier used in the RFE process when conducting the models. RF algorithm combines the ideas of bootstrap aggregating (bagging) and random feature selection, which is recognized as one of the best performing classifiers in the field of microarray analysis and other high-dimensional data (Li et al., 2021). The advantages of analyzing microbial data based on random forest have gradually emerged in forensic applications, including the estimation of postmortem interval (PMI) (Liu et al., 2020) and the time since deposition (TsD) (Smith et al., 2021).

This study aimed to investigate the potential use of microorganisms in identifying occupations in forensic science, which can be enhanced in several ways. First, the sample size was relatively small and focused on a single occupational group. To improve the validity of our findings, future research should encompass a larger sample size and individuals from various occupational groups within the same area, and even within the same family. Having a larger dataset would also be beneficial for studying the patterns of microbiota transmission between individuals on a larger scale (Brito et al., 2019; Valles-Colomer et al., 2023). Additionally, we utilized a limited number of annotation methods in this primary study. Applying the model construction approach introduced in section 2.5 to the data obtained from other annotation methods may be beneficial, such as read-based taxa annotation methods like Kraken2 (Wood et al., 2019), as well as various functional databases, including gene ontology (GO) database (Ashburner et al., 2000), eggNOG database (Powell et al., 2014), Pfam database (Mistry et al., 2021), SwissProt database (Bairoch, 1996), Carbohydrate-active enzymes (CAZy) database (Cantarel et al., 2009), or Cytochrome P450 Engineering (CYPs) database (Fischer et al., 2007). Furthermore, fresh saliva and fecal samples were collected and tested. However, in complex forensic settings, saliva often appears as stains and feces can exist in different forms, making it challenging to obtain fresh test materials. Therefore, future research should consider extracting and testing samples from simulated actual cases to further evaluate the applicability of oral and gut microbiota in forensic investigations.

## Data availability statement

Sequence data associated with this project have been deposited in the China National Center for Bioinformation (CNCB) database with project number PRJCA020943, which can be found at: https://ngdc.cncb.ac.cn/bioproject/browse/PRJCA020943.

## Ethics statement

The studies involving humans were approved by Medical Ethics Committee of Hebei Medical University. The studies were conducted in accordance with the local legislation and institutional requirements. The participants provided their written informed consent to participate in this study.

## Author contributions

SD: Conceptualization, Data curation, Investigation, Methodology, Writing—original draft, Software. GM: Conceptualization, Data curation, Formal analysis, Methodology, Software, Visualization, Writing—original draft. YL: Investigation, Validation, Writing—review & editing. GF: Investigation, Validation, Writing—review & editing. JS: Investigation, Validation, Writing—review & editing. LF: Investigation, Resources, Validation, Writing—review & editing. QW: Investigation, Resources, Writing—review & editing. TL: Formal analysis, Validation, Writing—review & editing. BC: Conceptualization, Supervision, Validation, Writing—review & editing. SL: Conceptualization, Funding acquisition, Methodology, Supervision, Writing—review & editing.
