# Supplementary material for: Preliminary exploratory research on the application value of oral and intestinal meta-genomics in predicting subjects' occupations–A case study of the distinction between students and migrant workers
Source: Front Microbiol. 2024 Feb 6;14:1330603. doi: 10.3389/fmicb.2023.1330603 (PMC10883652; doi:10.3389/fmicb.2023.1330603)
Supplement: Supplementary file 1 [file Data_Sheet_1.PDF]

Supplemental files of submission named "Preliminary exploratory research on the application value of oral and intestinal meta-genomics in predicting subjects' occupations–a case study of the distinction between students and migrant workers"

1 Supplemental Tables

1.1 Table S1

Table S1: Number of taxonomic detected in each sample

Sample Type	Occupation	Gender	Name	Kingdom	Phylum	Class	Order	Family	Genus	Species
Saliva	Student	Male	MSt01S	4	23	41	59	83	118	252
			MSt02S	4	27	50	78	118	180	382
			MSt03S	4	29	50	88	148	276	747
			MSt04S	5	107	165	269	464	1280	4486
			MSt05S	5	49	79	142	238	545	1740
			MSt06S	5	37	64	112	193	422	1212
			MSt07S	4	29	51	95	144	287	771
			MSt08S	5	98	187	348	654	1142	2102
			MSt09S	5	70	116	185	315	848	2952
			MSt10S	5	38	67	126	226	514	1638
			MSt11S	5	22	35	58	91	146	386
			MSt12S	5	45	83	142	238	489	1401
			MSt13S	5	62	100	160	266	598	1969
			MSt14S	4	50	77	124	198	429	1362
			MSt15S	5	27	56	101	170	253	561
			MSt16S	5	95	145	229	400	1077	3534
			MSt17S	5	74	118	186	313	801	2782
			MSt18S	6	64	105	179	305	796	2566
			MSt19S	5	37	69	124	239	552	1618
			MSt20S	4	27	50	78	125	222	698
		Female	FSt01S	5	24	40	70	101	164	469
			FSt02S	5	51	117	215	375	599	1104
			FSt03S	4	46	82	133	223	510	1648

Sample Type	Occupation	Gender	Name	Kingdom	Phylum	Class	Order	Family	Genus	Species
			FSt04S	5	46	82	133	209	400	1165
			FSt05S	5	68	109	178	298	786	2664
			FSt06S	4	25	46	80	128	208	622
			FSt07S	5	48	76	136	224	498	1580
			FSt08S	6	29	46	80	129	216	679
			FSt09S	5	38	63	108	166	340	1076
			FSt10S	4	52	82	140	230	508	1561
	Labourer	Male	MLa01S	6	106	161	259	440	1176	4005
			MLa02S	5	78	130	211	359	960	3252
			MLa03S	5	67	103	167	282	671	2002
			MLa04S	5	102	150	244	440	1142	3892
			MLa05S	6	123	184	289	500	1363	4531
			MLa06S	5	51	81	134	229	505	1547
			MLa07S	5	90	136	228	371	927	3036
			MLa08S	6	63	101	177	299	710	2322
			MLa09S	4	110	170	266	446	1169	3829
			MLa10S	6	54	97	152	229	419	1095
			MLa11S	5	128	203	317	571	1589	5813
			MLa12S	5	64	100	173	289	627	1892
			MLa13S	5	83	125	202	328	759	2358
			MLa14S	5	70	114	191	309	737	2326
			MLa15S	5	84	129	209	349	881	2900
			MLa16S	5	89	145	233	419	990	3172
			MLa17S	5	33	61	109	172	336	911
			MLa18S	6	82	125	202	329	771	2406
			MLa19S	5	57	96	166	263	588	1807
			MLa20S	6	127	188	292	496	1341	4646
Fecal	Student	Male	MSt01F	5	31	54	107	189	514	1964
			MSt02F	5	30	50	85	145	416	1916
			MSt03F	4	39	66	113	199	557	2294
			MSt04F	5	47	76	132	230	662	2757
			MSt05F	5	55	89	155	263	730	2914
			MSt06F	5	40	67	112	185	523	2400
			MSt07F	5	47	74	123	214	570	2460
			MSt08F	4	40	64	111	199	531	2239
			MSt09F	4	43	71	126	220	656	2885
			MSt10F	4	29	52	91	169	442	1928
			MSt11F	4	68	108	186	331	903	3403
			MSt12F	5	90	139	219	375	1032	3898
			MSt13F	4	39	65	110	190	511	2167
			MSt14F	4	40	67	116	197	557	2285
			MSt15F	4	86	146	243	413	1146	4166

Sample Type	Occupation	Gender	Name	Kingdom	Phylum	Class	Order	Family	Genus	Species
			MSt16F	4	34	61	111	198	561	2251
			MSt17F	4	23	39	75	130	309	1189
			MSt18F	4	39	65	116	198	579	2510
			MSt19F	4	64	96	164	278	772	3097
			MSt20F	6	45	76	133	226	636	2614
		Female	FSt01F	4	52	87	151	253	701	2775
			FSt02F	5	48	83	140	242	684	2715
			FSt03F	4	56	90	151	264	705	2785
			FSt04F	4	73	125	221	376	1043	4008
			FSt05F	4	62	100	161	287	805	3316
			FSt06F	4	36	62	103	179	456	1960
			FSt07F	5	34	56	86	151	453	1970
			FSt08F	4	49	83	142	246	676	2849
			FSt09F	4	47	78	131	215	511	1937
			FSt10F	4	77	128	220	379	1049	3893
	Labourer	Male	MLa01F	4	38	66	109	191	513	1982
			MLa02F	4	47	83	144	253	729	2931
			MLa03F	5	38	65	110	196	519	2158
			MLa04F	5	40	63	105	178	495	2280
			MLa05F	5	39	63	106	195	584	2633
			MLa06F	5	52	85	142	247	635	2405
			MLa07F	5	51	77	124	199	536	2221
			MLa08F	5	49	77	134	228	598	2297
			MLa09F	6	52	88	153	271	734	3050
			MLa10F	5	58	94	161	277	763	2963
			MLa11F	5	46	72	118	213	593	2512
			MLa12F	6	47	75	128	223	573	2217
			MLa13F	5	36	58	103	173	466	1922
			MLa14F	5	40	71	123	205	522	2023
			MLa15F	6	56	97	161	268	716	2820
			MLa16F	5	52	85	142	255	648	2485
			MLa17F	5	52	79	130	223	617	2528
			MLa18F	6	41	74	120	198	505	2209
			MLa19F	6	40	70	110	183	525	2268
			MLa20F	6	43	73	123	205	553	2257
Mean in all samples				4.83	54.78	89.82	150.57	256.53	643.79	2311.75
Total number in all sample				6	187	333	563	1110	3687	17005

1.2 Table S2

Table S2: Data set division

	Labourers		Students	
No.	Training set	Test set	Training set	Test set
1	MLa03	MLa01	MSt02	MSt01
2	MLa05	MLa02	MSt03	MSt07
3	MLa06	MLa04	MSt04	MSt08
4	MLa07	MLa08	MSt05	MSt12
5	MLa09	MLa16	MSt06	MSt13
6	MLa10	MLa17	MSt09	MSt16
7	MLa11		MSt10	
8	MLa12		MSt11	
9	MLa13		MSt14	
10	MLa14		MSt15	
11	MLa15		MSt17	
12	MLa18		MSt18	
13	MLa19		MSt19	
14	MLa20		MSt20	

1.3 Table S3

Table S3-1: Features selected in the final 12 models (part 1)

	Taxonomic		KEGG		CARD	
No.	Saliva	Fecal	Saliva	Fecal	Saliva	Fecal
1	*[Eubacterium] brachy*	*[Bacteroides] pectinophilus*	ko03022	ko04145	Fosfomycin	Aminoglycoside
2	*Acinetobacter baumannii*	*[Clostridium] dakarense*	ko03420		Rifamycin	Pleuromutilin
3	*[Eubacterium] infirmum*	*Youngiibacter fragilis*	ko04122		Disinfecting Agents And Antiseptics	Cephalosporin
4		*[Clostridium] aminophilum*	ko03030		Elfamycin	Sulfonamide
5		*[Clostridium] leptum*	ko04145		Antibacterial Free Fatty Acids	Carbapenem
6			ko03070		Peptide	Tetracycline
7			ko03060		Bicyclomycin	Bicyclomycin
8			ko03020		Macrolide	Aminocoumarin
9			ko00040		Cephamycin	
10			ko04120		Carbapenem	
11			ko03013		Aminoglycoside	
12			ko03410		Pleuromutilin	
13			ko03440		Diaminopyrimidine	
14			ko03018		Aminocoumarin	
15			ko00030		Mupirocin	

No.	Taxonomic: Saliva	Taxonomic: Fecal	KEGG: Saliva	KEGG: Fecal	CARD: Saliva	CARD: Fecal
16			ko00020		Tetracycline	
17			ko00010		Phenicol	
18			ko00051		Sulfonamide	
19			ko03430			

Table S3-2: Features selected in the final 12 models (part 2)

	VFDB		PHI-base		BacMet	
No.	Saliva	Fecal	Saliva	Fecal	Saliva	Fecal
1	VF0562	VF0569	PHI:850	PHI:1017/PHI:2067	znuB/yebI	znuC/yebM
2	VF0568	VF0567	PHI:852	PHI:1018/PHI:2042	znuA/yebL	zinT/yodA
3	VF0028	VF0566	PHI:881	PHI:881	zraS/hydG	zraS/hydG
4	VF0005	VF0571	PHI:1017/PHI:2067		zupT/ygiE	zupT/ygiE
5	VF0035	VF0573	PHI:849		zur/yjbK	zur/yjbK
6	VF0561	VF0572	PHI:851		znuC/yebM	zraR/hydH
7	VF0027	VF0568	PHI:1018/PHI:2042		zraR/hydH	
8	VF0039		PHI:862			
9	VF0025		PHI:824/PHI:6838			
10	VF0569		PHI:877			
11	VF0033		PHI:835			
12	VF0014					
13	VF0003					
14	VF0015					
15	VF0564					
16	VF0573					
17	VF0572					

2 Supplemental Figures

2.1 Figure S1

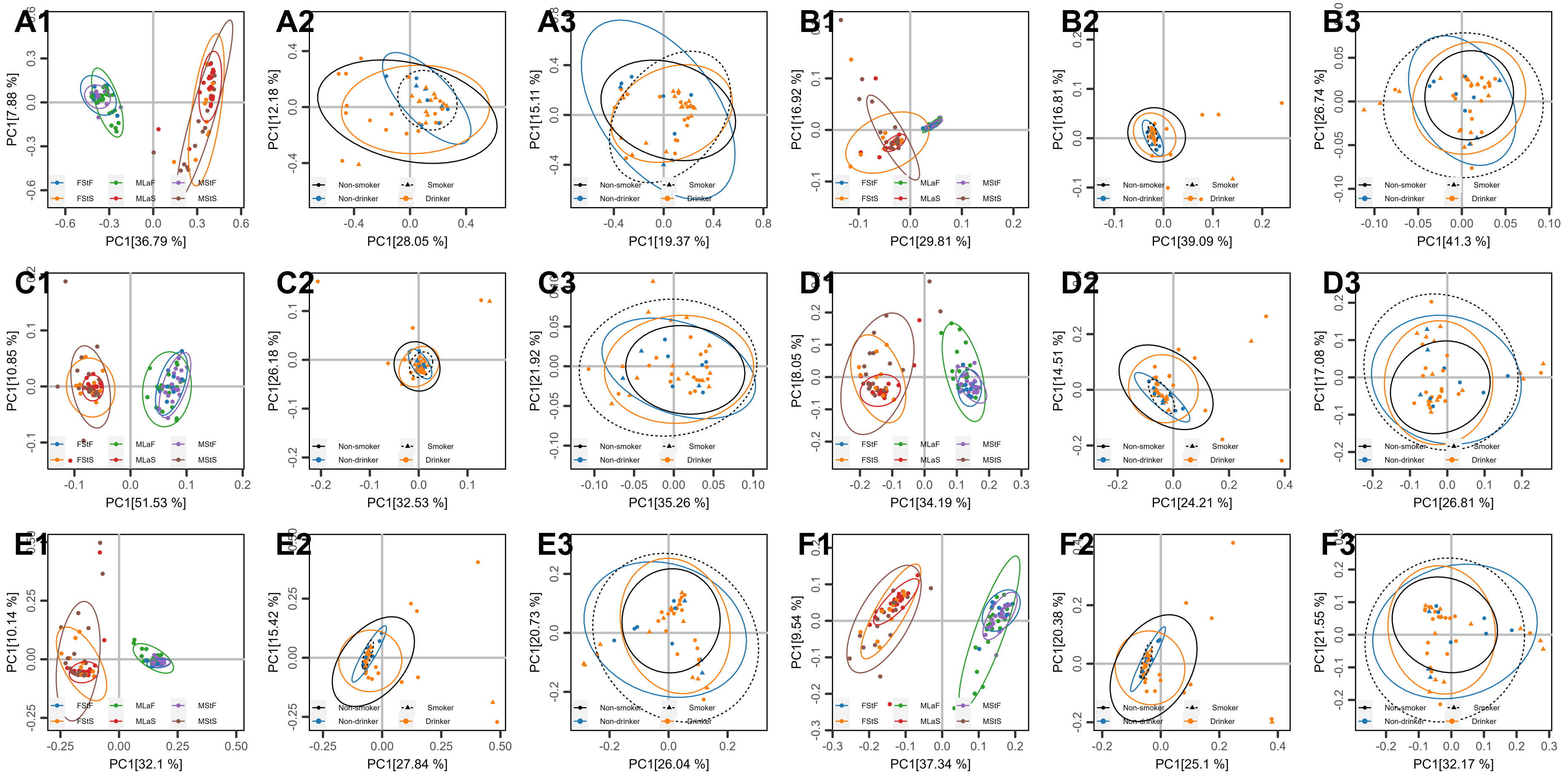

Figure S1: PCoA results based on 6 types of annotation methods: (A) Taxonomic; (B) KEGG; (C) CARD; (D) VFDB; (E) PHI-base; (F) Bacmet. Each sub-figure is formed by three parts: (1) PCoA results for all samples labeled with different colors based on gender, occupation and sample type; (2) PCoA results for male saliva samples divided with smoking (shape and line type) or drinking (color) status; (3) PCoA results for male fecal samples divided with similar method to (2).

3 Supplemental Files

3.1 File S1. R code of the RFE process

The RFE process in Algorithm 1 of the main text is performed in R (4.3.1) with the following code, which is provided as a separate file named “File_S1.R”.

```
#Library the package (line 1 of Algorithm 1)
library(randomForest)
library(zCompositions)
type<-data.frame(type=c("S","F"))
#Divide the 40 individuals into training and test sets with ratio of 7:3 (line 2 of Algorithm 1)
set.seed(123)
St_select<-sample(20,14)
set.seed(456)
La_select<-sample(20,14)
#Input basic data (line 3 of Algorithm 1)
basicdata<-read.csv("File_S2.csv",row.names = 1,header = TRUE)
#for each feature type do (line 4 of Algorithm 1)
for (k in 1:6) {
        #Extract basic data of corresponding feature type (line 5 of Algorithm 1)
        oridata<-basicdata[basicdata$method==k,2:81]
        oridata<-as.data.frame(t(oridata))
        group<-data.frame(Sample=rownames(oridata))
        group$group<-substr(group$Sample,1,2)
        group$Type<-substr(group$Sample,nchar(group$Sample),nchar(group$Sample))
        #for each sample type do (line 6 of Algorithm 1)
        for (j in 1:2) {
                #Extract basic data for the corresponding sample type (line 7 of Algorithm 1)
                filterdata<-oridata[group$Type==type$type[j],]
                group2<-group[group$Type==type$type[j],]
                #Filter out features not detected in more than 10 samples (line 8 of Algorithm 1)
                filterdata[filterdata>0]=1
                detectednumber<-as.data.frame(colSums(filterdata))
                data75<-oridata[group$Type==type$type[j],detectednumber>29]
                data75<-cmultRepl(data75,z.warning = 1)
                #Transform feature names into standard format to avoide error caused by feature names
                featuresnames<-data.frame(Number=paste(type$type[j],1:ncol(data75),sep = ""),features=colnames(data75))
                modelbasic<-cbind(data75,group2$group)
                colnames(modelbasic)=c(featuresnames$Number,’group’)
                modelbasic_St<-modelbasic[modelbasic$group==’St’,]
                modelbasic_La<-modelbasic[modelbasic$group==’La’,]
                # Divide the basic data into training and test sets (line 9 of Algorithm 1)
                training_set<-rbind(modelbasic_St[St_select,],modelbasic_La[La_select,])
                test_set<-rbind(modelbasic_St[-St_select,],modelbasic_La[-La_select,])
                #Conduct the first ‘‘bestmodel’’ (line 10 of Algorithm 1)
                bestmodel<-randomForest(as.factor(training_set$group)~.,data=training_set,importance=TRUE,ntree=100)
                #Evaluate the accuracy of ‘‘bestmodel’’ in training (accb1) and test (accb2) sets (line 11 of Algorithm 1)
                train_predict<-predict(bestmodel,training_set)
                compare_train<-table(training_set$group,train_predict,dnn=c(’Actual’,’Predicted’))
                test_predict<-predict(bestmodel,test_set)
                compare_test<-table(test_set$group,test_predict,dnn=c(’Actual’,’Predicted’))
                accb1=sum(diag(compare_train)/sum(compare_train))
                accb2=sum(diag(compare_test)/sum(compare_test))
                #Record the best accuracy of each round and the features in ‘‘bestmodel’’
```

```
        bestaccchange<-data.frame(N=ncol(data75):1,Train=0,Test=0)
        bestfeatures<-importance_features<-data.frame(averageimportance=rep(0,ncol(test_set)-1),rank=0)
        rownames(bestfeatures)=rownames(importance_features)<-colnames(test_set)[1:ncol(test_set)-1]
        #Set N = the count of the total number of the remain features (line 12 of Algorithm 1)
        N<-ncol(data75)
        #While N>0 (line 13 of Algorithm 1)
        while (N>0){
            import<-as.data.frame(matrix(nrow = nrow(importance_features),ncol = 100))
            rownames(import)<-rownames(importance_features)
            Accuracydata<-data.frame(No=1:100,acctrain=0,acctest=0)
            #for i=1:100 do (line 14 of Algorithm 1)
            for (i in 1:100) {
                #Conduct a template model with all N features based on the training set (line 15 of Algorithm 1)
                tempmodel<-randomForest(as.factor(training_set$group)~.,data=training_set,importance=TRUE,ntree=100)
                #Evaluate the importance of each feature in the model (line 16 of Algorithm 1)
                import[,i]=tempmodel$importance[,3]
                #Evaluate the accuracy of each model in training (acci1) and test (acci2) sets (line 17 of Algorithm 1)
                train_predict<-predict(tempmodel,training_set)
                compare_train<-table(training_set$group,train_predict,dnn=c('Actual','Predicted'))
                test_predict<-predict(tempmodel,test_set)
                compare_test<-table(test_set$group,test_predict,dnn=c('Actual','Predicted'))
                Accuracydata$acctest[i]=sum(diag(compare_test)/sum(compare_test))
                Accuracydata$acctrain[i]=sum(diag(compare_train)/sum(compare_train))
                #If acci1+acci2>=accb1+accb2 then (line 18 of Algorithm 1)
                if (Accuracydata$acctrain[i]+Accuracydata$acctest[i]>=accb1+accb2) {
                    # Replace ‘‘bestmodel’’, accb1 and accb2 with the current model (line 19-21 of Algorithm 1)
                    bestmodel<-tempmodel
                    accb1<-Accuracydata$acctrain[i]
                    accb2<-Accuracydata$acctest[i]
                    bestfeatures<-importance_features
                }
            }
            #Rank the features with the average of the mean decrease accuracy in the 100 models (line 24 of Algorithm 1)
            Accuracydata$sum<-Accuracydata$acctrain+Accuracydata$acctest
            Accuracydata<-Accuracydata[Accuracydata$sum==max(Accuracydata$sum),]
            bestaccchange$Train[bestaccchange$N==N]<-Accuracydata$acctrain[1]
            bestaccchange$Test[bestaccchange$N==N]<-Accuracydata$acctest[1]
            importance_features$averageimportance=rowSums(import)/100
            importance_features$rank<-order(importance_features$averageimportance,decreasing=TRUE)
            N=N-1 #(line 25 of Algorithm 1)
            #Eliminate the lest valuable feature (line 26 of Algorithm 1)
            importance_features<-importance_features[importance_features$rank<=N,]
            selected_features<-rownames(importance_features)
            training_set<-training_set[,c(selected_features,'group')]
            test_set<-test_set[,c(selected_features,'group')]
        }
        #Output the best model of the corresponding sample type and feature type (line 28 of Algorithm 1)
        bestfeatures$name<-featuresnames$features[which(featuresnames$Number %in% rownames(bestfeatures))]
        besttrain<-rbind(modelbasic_St[St_select,c(rownames(bestfeatures),'group')],
        modelbasic_La[La_select,c(rownames(bestfeatures),'group')])
        besttest<-rbind(modelbasic_St[-St_select,c(rownames(bestfeatures),'group')],
        modelbasic_La[-La_select,c(rownames(bestfeatures),'group')])
        save(bestaccchange,bestmodel,bestfeatures,besttrain,besttest,accb1,accb2,
        file=paste('File_S3_',2*k-2+j,'.RData',sep=''))
```

```
103         }
104  }
```

3.2 File S2. Abundance data used in the RFE process

This file is separately provided as a .csv files, which is needed by the code in File S1.

3.3 File S3. The 12 best models

This file is separately provided as a .zip file, containing 12 .Rdata files generated by the code in File S1. Each .Rdata file is named as "File_S3_X.Rdata" where X ranged from 1 to 12, the correspondence of which with the model details is provided in section 3.3 of the main text. Information in these files can be loaded in R with function *load("File_S3_X.Rdata")*. After being loaded, 7 vectors can be seen:

- **bestmodel**: The best model constructed through RFE process;
- **bestfeatures**: Features involved in the final model, two types of names are provided: *i*) the actual name of the features, and *ii*) the "standard" names of them transformed in the RFE process to avoid errors by the feature names (see line 30-31 of the code in File S1);
- **besttrain**: Relative abundance data of the features in the final model in training set;
- **besttest**: Relative abundance data of the features in the final model in test set;
- **accb1**: Accuracy of the final model in training set;
- **accb2**: Accuracy of the final model in test set;
- **bestaccchange**: The change of the best accuracy of each round of RFE process.
